# Prenatal Presentation of Medulloepithelioma: Case and Literature Review

**DOI:** 10.7759/cureus.5018

**Published:** 2019-06-27

**Authors:** Nidhi Arora, Chanchal Ahmad, Arpit Gupta, Nitin Ghonge, Anita Kaul

**Affiliations:** 1 Fetal Medicine, Indraprastha Apollo Hospitals, Delhi, IND; 2 Fetal Medicine, Madhukar Rainbow Children's Hospital, Delhi, IND; 3 Neonatology, Metropolitan Hospital Center, New York, USA; 4 Radiology, Indraprastha Apollo Hospitals, Delhi, IND

**Keywords:** ultrasound, fetal mri, intracranial tumor, prenatal diagnosis, medulloepithelioma

## Abstract

Congenital brain tumors (CBTs) are extremely rare and account for only 0.5%-1.9% of all pediatric brain tumors. Medulloepithelioma is one of the rare tumors with an incidence of about 1% among all CBTs with a very dismal prognosis and typically diagnosed at the median age of 24 months. The objective is reporting medulloepithelioma presenting in the intrauterine period with very few prior cases being reported in the prenatal period, and to add to the limited existing literature on medulloepithelioma. We present a rare case of medulloepithelioma referred to us in the antenatal period at 27 weeks and subsequently causing intrauterine fetal demise. Prenatal MRI of the fetal brain and postnatal histopathological findings on autopsy were suggestive of intracranial medulloepithelioma.

## Introduction

Congenital brain tumors (CBTs), defined as tumors presenting within 60 days after birth, are extremely rare and account for only 0.5-1.9% of all pediatric brain tumors [1-3]. The most frequently reported tumors are teratomas (63%) and gliomas (30%) [4, 5]. Embryonal tumors (7%) account for rest of the CBTs. Most of the time intracranial tumors (ICTs) are detected incidentally on routine fetal imaging as a mass with or without hydrocephalus with macrocephaly as a late feature. Prognosis is usually guarded with an overall neonatal survival rate of 28% [6]. Medulloepithelioma is one of the rare embryonal tumors with an incidence of about 1% among all CBTs with a very dismal prognosis [6]. Making a definitive diagnosis requires histopathology after tissue biopsy which is very difficult in the fetal period. Therefore, the diagnosis is usually made on the basis of fetal sonogram and fetal MRI findings [7, 8].

We here present a case of medulloepithelioma which was initially diagnosed with glioma based on fetal imaging findings but later on diagnosed as medulloepithelioma based on tissue histopathology. Medulloepithelioma tumors are rare with an average age of two years at presentation with very few cases diagnosed in the neonatal period [6, 9]. Most of the information on medulloepithelioma is based on the prior reported cases. The objective of reporting this case is to add to the very limited information on the medulloepithelioma presentation especially in the fetal period and its findings in the fetal imaging (ultrasound and MRI). We will also do a brief review on medulloepithelioma based on previously reported cases [9-14].

## Case presentation

A 35-year-old gravida three and para one female was referred to the fetal medicine department in view of an intracranial mass detected on routine growth scan at 27 weeks. Anomaly scan at 19 weeks was normal. The mother gave no history of fever with rash, bleeding disorders, radiation exposure, drug intake or substance abuse. She was not hypertensive or diabetic and was not on any medication apart from iron and calcium supplementation. There was no personal or family history of malignancy in either partner.

**Figure 1:**
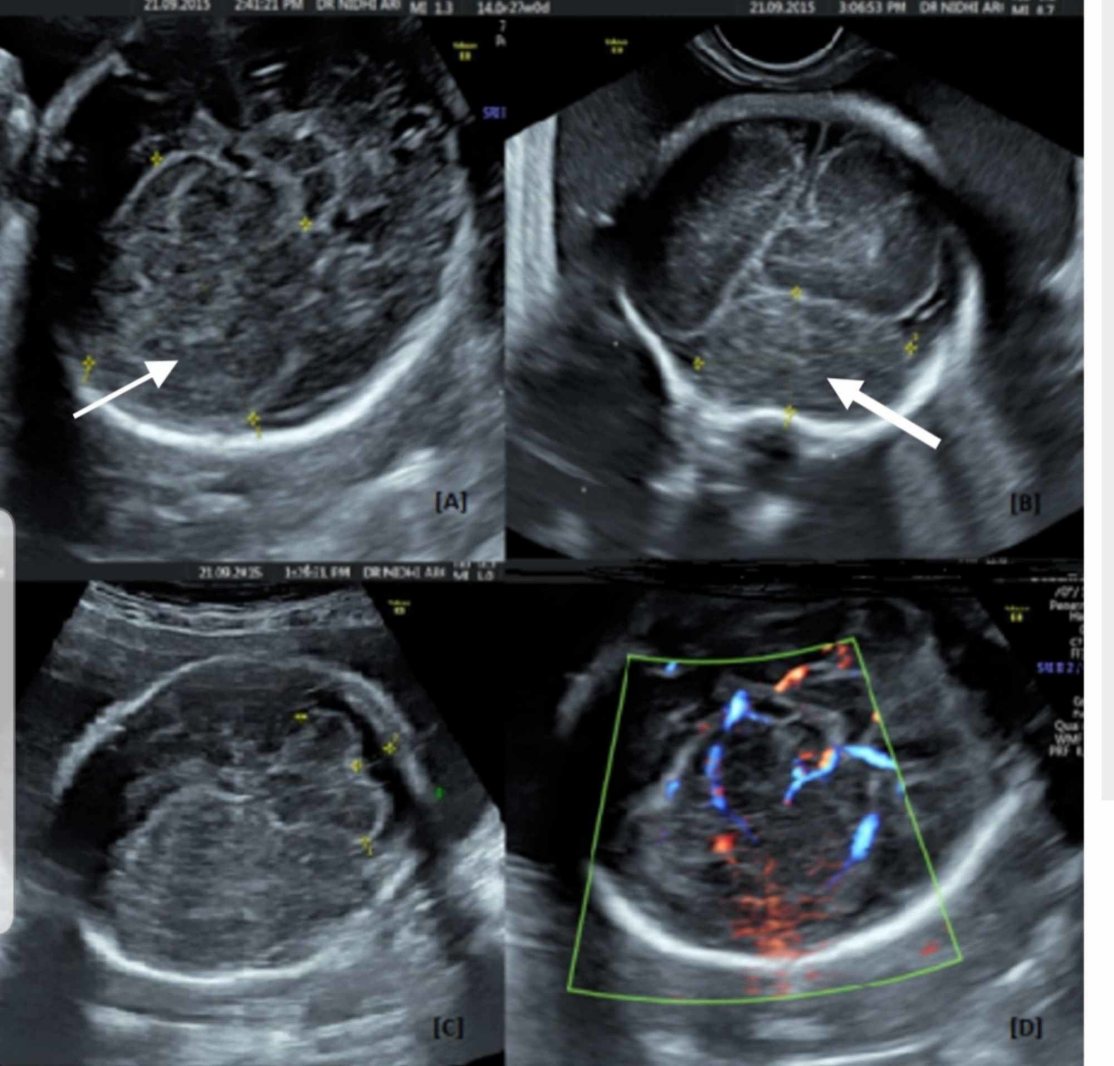
Ultrasound images of the intracranial mass. (A) 2D image showing homogenous mass in right frontal lobe in transthalamic plane (white arrow). (B) Coronal plane (thick white arrow). (C) Normal posterior fossa. (D) Color Doppler showing feeding vessels into the mass.

Ultrasound was done using Voluson E-Radiance (GE Healthcare, Milwaukee, WI) equipped with a convex 4-8 MHz abdominal probe, and 6-12 MHz endovaginal probe. Two-dimensional ultrasound (Figure 1A-1C) showed an intracranial mass in the fetal right frontal lobe measuring 4.5 x 3.8 x 3 cm with echogenicity similar to the adjacent normal brain. The mass was crossing the midline. A detailed neurosonogram was done. There was no associated ventriculomegaly. The posterior fossa structures were normal. Transvaginal ultrasound was done to confirm the findings and to determine the spread of the lesion. On color Doppler, feeding vessels were identifiable (Figure 1 D). There was no other structural abnormality. Fetal echocardiography was normal. Fetal growth was within the normal range for gestation. There was polyhydramnios (amniotic fluid volume above the 95th centile). Diagnosis of an isolated intracranial mass was made.

**Figure 2:**
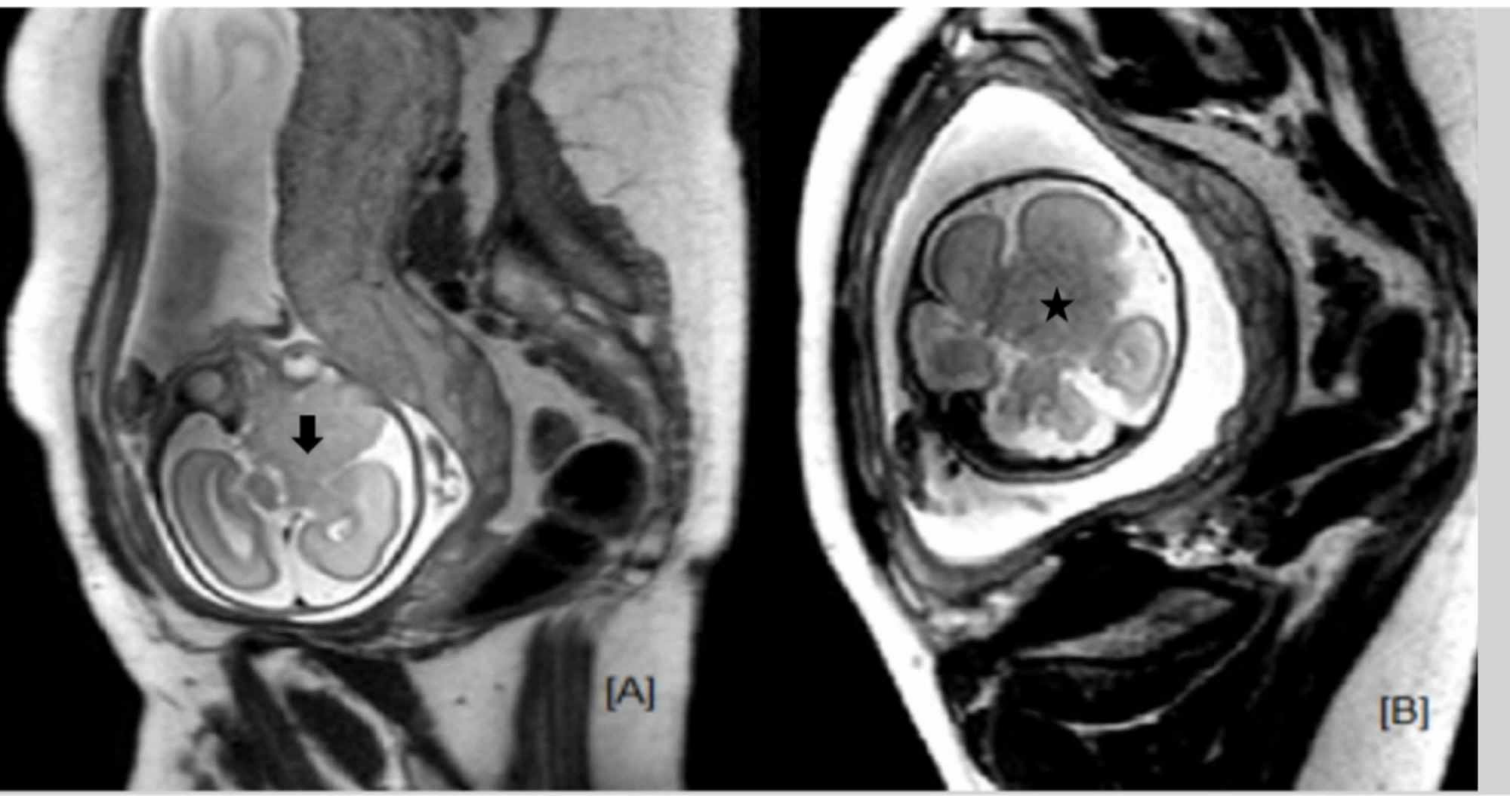
Fetal MRI brain. (A) Axial T2WI HASTE image showing ill-defined round mass (arrow) in left frontal lobe. (B) Coronal image showing inferior extent of the lesion into the thalami (star). HASTE: Half-Fourier acquired single shot turbo spin echo

Fetal MRI was performed on a 3 Tesla mode, Philips 3T scanner. T2-weighted axial, coronal and sagittal images were acquired, along the fetal planes using half Fourier acquired single shot turbo spin echo (HASTE) sequences for fetal central nervous system (CNS). Fetal MRI showed a focal intra-axial mass lesion in the right frontal location. Posterosuperiorly the extent was up to right basal ganglia and thalamic region with indentation over the third ventricle. The lesion was not seen separate from the crus cerebri. There was compression over the septum pellucidum, which was displaced to the left by 2-3 mm. The lesion measured 4.8 cm x 4.0 cm x 2.8 cm in antero-posterior, transverse, and craniocaudal dimension, respectively. It was hypointense on T2W imaging as compared to white matter and showed hypointense to isointense signal on T1W images. The fetal ventricular system showed extrinsic compression, mainly over the right lateral ventricle and the third ventricle. The fourth ventricle was not dilated. The posterior fossa structures were normal. No definite signs of proptosis or intraorbital extension were seen (Figure 2). A provisional diagnosis of a glioma, possibly of hypothalamic/thalamic origin was made.

**Figure 3:**
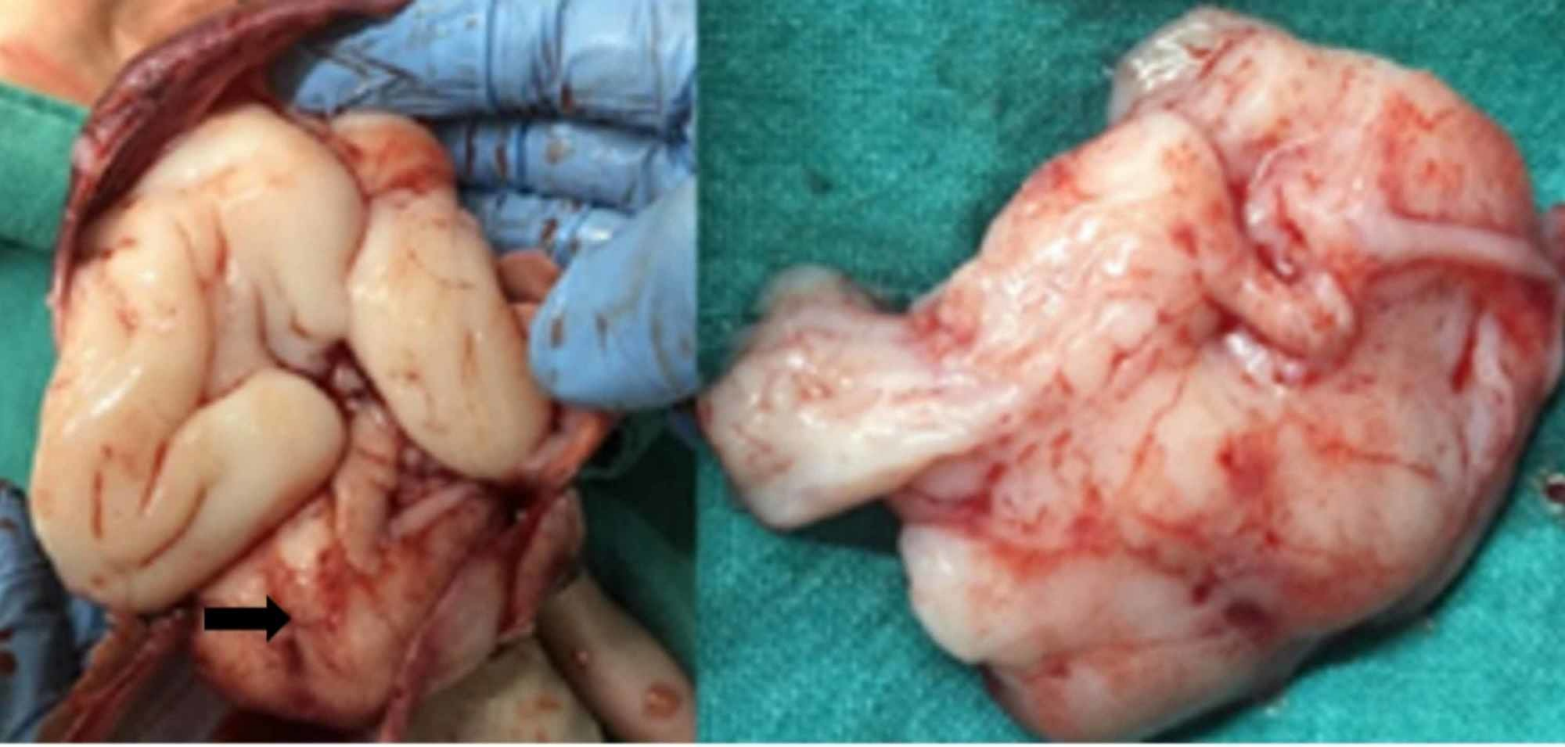
Gross examination of the tumor. (A) Solid homogenous mass arising from right frontal lobe (arrow). (B) Resected tumor mass.

**Figure 4:**
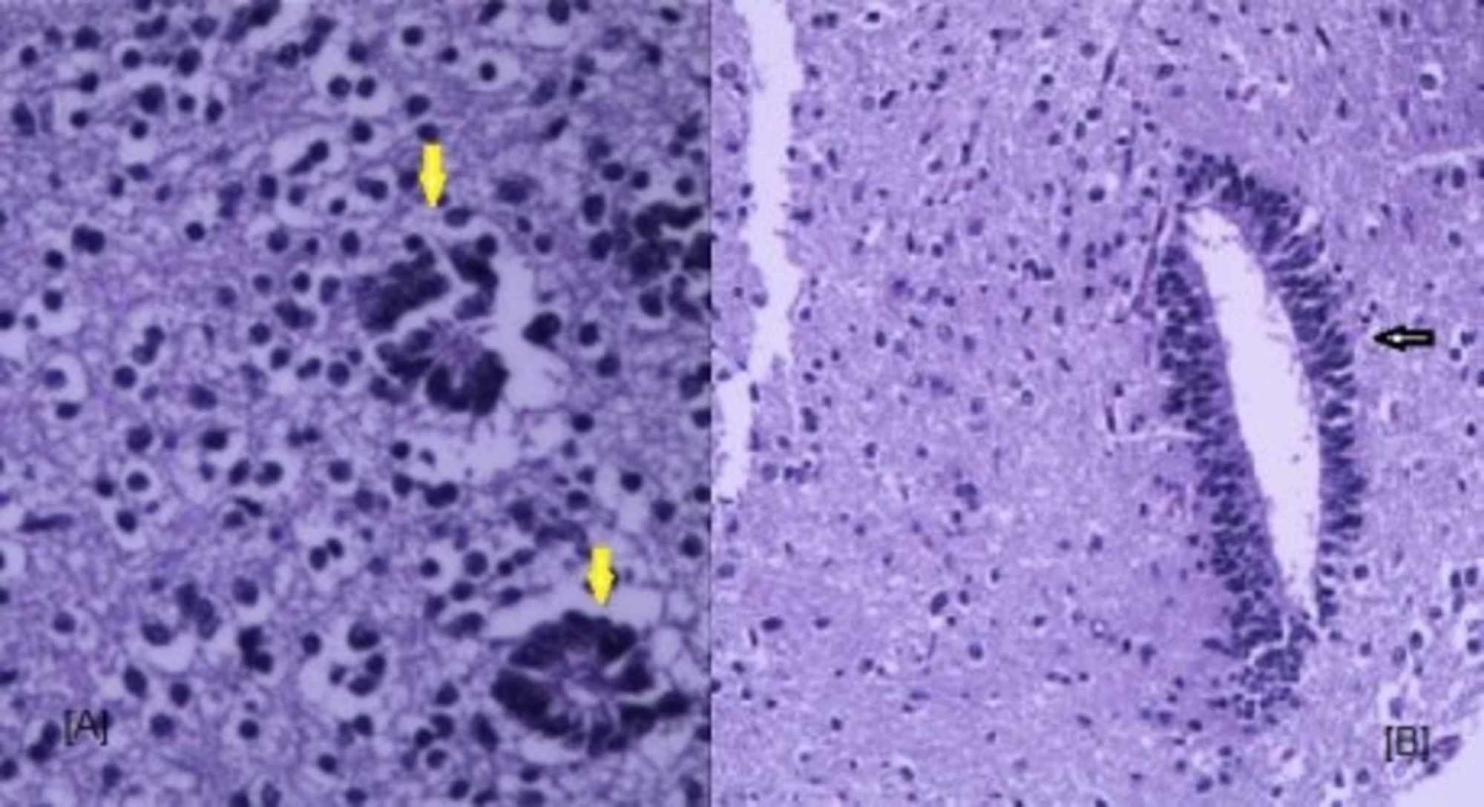
Histopathology of the resected mass. (A) 20X magnification - Dyscohesive sheets of cells forming rosettes (yellow arrows) at focal places. The rosettes show well-defined empty small lumen bordered by a thick membrane. (B) 10X magnification - Scattered few elongated canals (black arrow) lined by stratified columnar type epithelium at the interface with adjacent brain tissue.

A joint consultation with the neonatologist, pediatric neurosurgeon, and pediatric neurologist was done. The couple was counseled regarding the expected poor prognosis of antenatally diagnosed intracranial tumors in view of its imaging findings. The timing of delivery and the need for close follow-up was also discussed. Follow-up ultrasound one week later at 28 weeks showed no fetal cardiac activity. Induction of labor was done and a stillborn male fetus weighing 1300 grams was delivered vaginally. The couple consented for fetal autopsy. The external examination was normal. There were no dysmorphic features. On autopsy, there was a large, homogenous, right-sided frontal tumor extending to the base of the skull. There were no necrotic areas and no hemorrhages (Figure 3). The cerebellum was normal. Histopathology gave the unexpected diagnosis of medulloepithelioma which is a rare tumor of embryonal origin (Figure 4). The histopathology showed a tumor, composed of nests, tubules, and trabecular arrangement of malignant cells, lined by pseudostratified epithelia, resembling primitive neural tube, sheets of poorly differentiated cells with hyperchromatic nuclei. Immunohistochemistry was positive for synaptophysin, and vimentin suggestive of medulloepithelioma.

## Discussion

Medulloepitheliomas are very rare embryonal tumors with an incidence of 1% to 1.5% among all CBTs [6]. They were earlier described under the umbrella of primitive neuroendocrine tumors (PNETs), but as per the latest 2016 WHO classification of brain tumors, they fall under the category of embryonal tumors with multilayered rosettes (ETMR) or embryonal tumors with abundant neuropil and true rosettes (ETANTR) [15].

Many of these rare tumors display amplification of the C19MC region on chromosome 19 (19q13.42) and hence described as ETMR C19MC-altered. In the absence of C-19 amplification, they can be described as ETMR NOS, and if the histopathology is suggestive of medulloepithelioma, then should be named as medulloepithelioma. However, significant proportions of medulloepithelioma have not shown C19MC alterations [15]. The main histopathology description for this tumor is the papillary, tubular, or trabecular arrangement of pseudostratified neuroepithelium resembling an embryonic neural tube. Others are multi-layered rosettes and evidence of multiple lines of differentiation including neuronal, glial and mesenchymal elements [1,6,14]. Majority of these tumors are immunoreactive for vimentin, and sometimes are immunoreactive for glial and neuronal antigens. Regardless of the change in the classification, medulloepitheliomas are still considered as highly malignant grade IV tumor [15].

It was first described by Bailey and Cushing in 1926 [11]. It is a rare malignant tumor of childhood with an average age at diagnosis is two years. Only very few congenital patients have been reported [6,14]. There is a very limited data on medulloepithelioma identified as early as 27 weeks post-conceptual age, and causing fetal demise. Cassart et al. in an extensive review reported 27 cases of fetal intracranial tumors over a period of 14 years [16]. They retrospectively analyzed imaging and clinical findings in 27 cases of fetal intracranial tumors assessed by ultrasound and MR imaging followed with histologic confirmation. They diagnosed 15 germinal tumors, four glial tumors, two craniopharyngiomas, and three hamartomas. No PNET was diagnosed. Isaacs reported only three cases of medulloepithelioma out of 250 cases (1.3%) in one of the extensive literature review of brain tumors diagnosed in a fetal and neonatal period [6]. All of the three cases presented with macrocephaly with some neurological signs upon presentation with no cases of stillbirth.

Majority of medulloepithelioma carries a bad prognosis with a dismal survival of patients. Molloy et al. reported a case series of eight cases of CNS medulloepithelioma diagnosed in their center over a period of 14 years [9]. The reported incidence of medulloepithelioma was eight out of 800 cases (1%, similar to what reported by Isaacs) of primary brain tumors diagnosed by histopathology. The mean age of diagnosis ranged from six to 52 months with a median age of 24.5 months. Six out of eight cases underwent surgery with extensive resection of brain tissue followed with adjuvant therapy postoperatively (radiation therapy or chemotherapy). Six patients died within three days to 20 months post-diagnosis. Two patients survived but with significant neurological impairment.

In the same case series by Molloy et al., the majority of medulloepithelioma were either hypointense (four of five cases) or isointense (one of five cases) on T1-weighted MR imaging. The tumors were well circumscribed, mildly heterogeneous mass with no evidence of hemorrhage at presentation. The intracranial tumor mass, in this case, was also mildly hypointense on T1-weighted imaging with no evidence of hemorrhage upon presentation (at 27 weeks). On histopathology, the mass had a characteristic appearance of medulloepithelioma as described above. The C19MC amplification test was not performed in this case. However, that does not rule out the diagnosis of medulloepithelioma, since not all medulloepithelioma tumors manifest C19MC amplification (WHO new classification) [15].

A rare tumor, intraorbital medulloepithelioma primarily in the ciliary body is histologically similar to an intracranial tumor. However, it carries a better prognosis and exhibits less malignant course. Patients with intraorbital medulloepithelioma, treated only with enucleation achieve excellent long-term survival compared with the dismal survival of patients with intracranial medulloepitheliomas. The exact reason for better prognosis in the intraorbital location is still unexplained [17].

The fetus, in this case, had normal fetal anatomy sonogram at 19 weeks but noted to have a large intracranial mass at 27 weeks measuring 4.8 cm x 4.0 cm x 2.8 cm. Subsequent follow-up one week later revealed stillbirth. The small time period from undetected mass at 19 weeks to a relatively huge mass at 27 weeks and subsequent unexplained intrauterine fetal demise may indicate an aggressive nature and the lethality of intracranial medulloepithelioma in the fetal period. On average, the gestational age at diagnosis is 27 weeks for teratomas, 21 weeks for hamartomas, and 34 weeks for gliomas [16, 18]. No information is available on the relevant gestational age for the other types of CBTs including medulloepithelioma.

## Conclusions

Medulloepithelioma is a rare tumor with a general perception of it being an early childhood tumor. The objective of reporting this case is to add to the limited literature about the medulloepithelioma especially its fetal presentation and fetal imaging findings. It may guide the clinicians to consider medulloepithelioma as a differential while approaching the family. However, the chances of intracranial fetal mass being a medulloepithelioma will be still very small looking at the overall incidence of only 1% among all ICTs. Looking at the overall grim prognosis of medulloepithelioma and similar embryonal tumors, it is important to provide detailed information to family and involve them in the decision-making process along with providing support to the family to determine the course of treatment after the diagnosis.
